# Should a Multigene Signature be Used in all Luminal Early Breast Cancers

**DOI:** 10.3389/fonc.2019.00454

**Published:** 2019-06-04

**Authors:** Nawale Hajjaji, Yves Marie Robin, Jacques Bonneterre

**Affiliations:** ^1^Breast Cancer Department, Oscar Lambret Cancer Center, Lille, France; ^2^Prism Laboratory, Inserm, University of Lille, Lille, France; ^3^Pathology Department, Oscar Lambret Cancer Center, Lille, France; ^4^School of Medicine, University of Lille, Lille, France

**Keywords:** multigene signature, PAM50, luminal breast cancer, risk of recurrence, clinical risk

## Abstract

**Background:** Multigene signatures refine the risk of recurrence and guide adjuvant chemotherapy decision in luminal breast cancers. The decision to perform the assay is highly variable among oncologists. In order to guide the appropriate clinical group in whom to perform a genomic signature, our study analyzed in a homogeneous cohort which clinical risk groups triggered the use of the PAM50-based signature and their concordance with the genomic risk.

**Methods:** A real life cohort of 222 early breast cancer patients with hormone receptor positive and HER2 negative disease had a commercial PAM50-based assay (Prosigna®) performed at our institution. The assay provided the risk group, the 10-year risk of distant recurrence and the intrinsic molecular subtype of breast cancer.

**Results:** Based on nodal involvement, Ki67, tumor grade, mitotic index, and tumor size, no clinical pattern could identify a specific genomic risk group. The discordance with the genomic risk was high in patients with clinical low risk tumors, both in node negative and node positive patients. Up to 60% of them had a 10% or more risk of distant recurrence. Moreover, we identified a subgroup of luminal A tumors with a high genomic risk of recurrence. Genomic risk and intrinsic subtype were strong determinants of chemotherapy decision.

**Conclusions:** Clinical profiles could not reliably identify genomic risk groups and guide the decision to use a multigene signature. Significant discordance with the genomic risk was observed within low clinical risk and luminal A tumors.

## Introduction

Luminal breast cancer has a wide range of outcomes not fully predicted by clinical or pathological features. Multigene signatures are used to refine the risk of distant recurrence, and guide adjuvant chemotherapy decision (1–3). These signatures are intended as a prognostic tool for postmenopausal women with early-stage breast cancer, after surgery, when tumors are hormone receptor-positive and HER2 negative.

There is no specific guidance regarding when to use a multigene assay. It is usually recommended in equivocal clinical situations in both node negative or node positive patients. However, this covers a wide and heterogeneous spectrum of clinical cases. The decision to use or not a multigene assay depends on the level of risk perceived by the oncologist. This estimation is based on clinical and pathological factors, mainly age, tumor size, nodal involvement, grade, Ki67, mitotic index, and hormone receptors (3, 4). However, the integration and interpretation of these factors in the decision process is highly variable among oncologists, which could impact patients' access to these assays. There is a need to rationalize the indications to use or not a multigene signature, especially given their financial burden.

The study aims were to analyze in a homogeneous real life cohort (i) which clinical risk groups triggered the use of the PAM50-based Prosigna® assay, and (ii) their concordance with the genomic risk. This genomic signature is among the commercialized multiparameter prognostic tests. It provides an estimate of the 10-year risk of relapse and the breast cancer intrinsic molecular subtype (5–8).

## Patients and Methods

### Patient Population

A population of early breast cancer patients with hormone receptor positive and HER2 negative tumors, all treated at our institution, whose risk of relapse was estimated using the commercial PAM50-based assay Prosigna® was identified through our clinical database. Two cohorts of patients tested with Prosigna® were available and pooled totalizing 222 cases: a cohort of 159 breast cancer cases (cohort 1) tested prospectively since the assay was commercialized in France, from June 2016 to August 2018, and a retrospective cohort of 63 cases with a clinical intermediate risk of relapse, tested for research purposes (9). Their characteristics are detailed in Supplementary Table 1. Additionally, the records of all the patients with HR+ HER2 negative tumors and less than 4 positive nodes not tested with Prosigna® and available in our database (initiated in 2016) during the same period as cohort 1 were analyzed. Treatment decision was collected and analyzed in cohort 1 (tested prospectively with Prosigna®). The study was approved by our institutional review committee and all procedures performed in this study were in accordance with the ethical standards of our institutional research committee. Informed consent was obtained from all individual participants.

### PAM50-Based Prosigna® Assay

Formalin-fixed, paraffin embedded blocks from the original tumors were collected to delineate an area with >70% of cancer cells for RNA extraction. Analyses were performed using the nCounter automated platform (nanoString® Technologies, Seattle, WA) following the manufacturers' instructions. The ROR (risk of recurrence) score was calculated for each sample using the expression profile plus tumor size to classify the tumor within a risk category: genomic low, intermediate or high risk. The test also provided an estimate of the 10-year risk of distant recurrence (in %). Each tumor sample was assigned to an intrinsic molecular subtype of breast cancer (luminal A, luminal B, HER2 enriched, or basal like) based on PAM50 subtype prediction.

### Clinical and Histological Data

Clinical and histopathological parameters were collected from patients' records. Immunohistochemical (IHC) staining was performed for estrogen receptor (ER) and progesterone receptor (PR) expression, HER2, and Ki67. The Allred score was used to evaluate ER and PR expression (10). Hormone receptor expression was defined as positive if ≥ 10% of the cells were stained positive. HER2 scoring followed ASCO/ACP guidelines (11). Scores of 0 and 1 were considered negative for HER2 expression and a score of 3 positive. FISH assay was performed for HER2 scores of 2. Histological grading was performed according to the Nottingham protocol (12). The guidelines of van Diest et al. were applied for the correct identification of mitotic figures (13). Ki67 assessment by IHC followed international recommendations (14). The Ki67 score was expressed as the percentage of positively stained cells among the total number of invasive cells in the area scored. Ki67 cut offs in our practice were 14% and 20% based on the 2013 St. Gallen recommendations (15, 16).

### Statistical Analysis

Statistical analyses were performed with the Graphpad Prism 8 software (GraphPad Software, USA). A Mann Whitney or Kruskal Wallis test was used to analyze the association between the ROR score or the risk of recurrence and tumor grade, mitotic index, Ki67, tumor size and nodal status. A Spearman test was used to explore the correlation between continuous variables and the PAM50 ROR score or risk of recurrence. A Chi2 test was used to compare categorical variables. *P* < 0.05 was considered significant.

## Results

### Cohort Characteristics

The genomic signature was performed in 222 cases. Patients and tumor characteristics were described in Table 1. Median patient age was 59 years; tumor size was T1 (≤2 cm) in 46% of the cases. Patients were node negative in 51% of the cases, and 84% were grade 2. Ki67 was <14% in 31% of the cases, and >20% in 34% of the cases. Mitotic index was classified as 1 in 61% of the cases. Among the 222 cases, the genomic signature classified 42 cases (19%) in the low risk group, 99 cases (45%) as intermediate risk and 81 cases (36%) as high risk. In genomic low risk tumors, 85% were luminal A and 15% were basal. In genomic intermediate risk tumors, 76% were luminal A and 20% luminal B. In genomic high-risk tumors, 31% were luminal A and 69% were luminal B.

**Table 1:** Patients and tumor characteristics.

	***N***
Age (median, range)	59 (29–81)
<50 years	51
≥50 years	171
Body Mass Index (median, range)	25.75 (16.96–54.43)
Tumor size	
T1	102
T2	102
T3	18
Lumpectomy	136
Mastectomy	86
Axillary exploration	
Sentinel lymph node	133
Axillary dissection	86
None	3
Histology	
Ductal	155
Lobular	43
Other	24
Grade	
1	20
2	187
3	15
Ki67	
<14%	68
14–20%	77
>20%	76
*n*	1
Mitotic index	
1	136
2	74
3	11
*n*	1
Estrogen receptor positive	216
Progesterone receptor positive	184
HER2 status negative	222
Nodal involvement	
0	113
1	63
2	36
3	10

### Clinical Profiles Triggering the Use of the Genomic Signature

**Figure 1:**
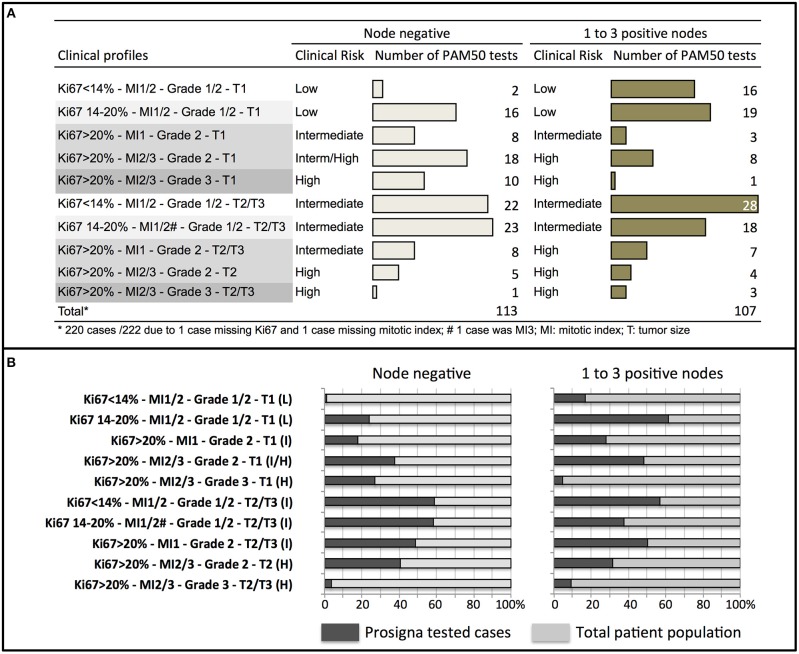
Distribution of clinical profiles and clinical risk groups in patients tested with Prosigna® in node negative or node positive (1–3 nodes) patients **(A)**, and proportion (in%) of cases tested with Prosigna® in the total patient population treated during the same period of time than cohort 1 **(B)**. H, high clinical risk; I, intermediate clinical risk; L, low clinical risk.

The main prognostic factors used at our institution to discuss whether a genomic test should be performed were: nodal involvement (0 to 3 positive nodes), three proliferative markers (Ki67, tumor grade and mitotic index), and tumor size. These factors were integrated into clinical profiles and a clinical risk estimate was attributed to each profile as per our practice. The number of Prosigna assay performed according to each clinical risk profile was detailed in node negative and in 1 to 3 node positive cases (Figure 1A). Among the tumors tested, 58% had a clinical intermediate risk, but 24% were clinical low and 18% clinical high-risk tumors.

Four hundred eighty-three cases of hormone receptor positive HER2 negative early breast cancer with less than 4 positive nodes were treated during the same period of time than cohort 1 at our institution (including cases tested and not tested with Prosigna). The proportion of clinical profiles tested with Prosigna was analyzed according to the total patient population (Figure 1B). In node negative patients, the clinical profiles that triggered the genomic signature were mostly large tumors (T2/T3) with low or discordant proliferative markers. In node positive patients, the same clinical profiles were observed and additionally small tumors (T1) with intermediate or discordant proliferative markers.

### Relationship Between Clinical Factors and Genomic Risk

**Figure 2:**
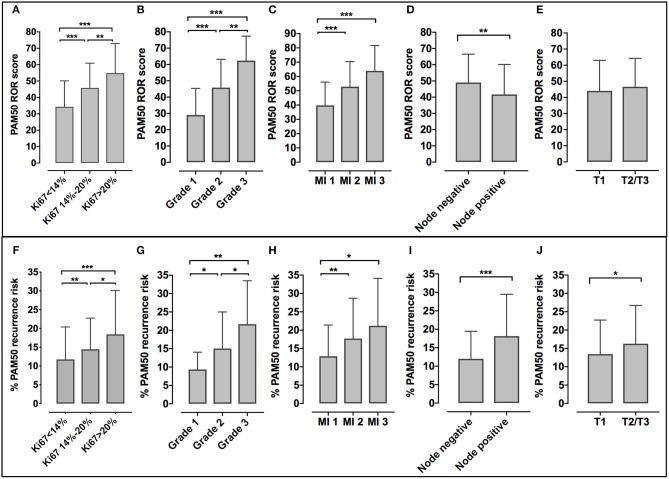
Relationship between the ROR score or the PAM50 risk of recurrence and Ki67 **(A,F)**, tumor grade **(B,G)**, mitotic index (MI) **(C,H)**, nodal status **(D,I)**, and tumor size (T1 or T2/T3) **(E,J)**. Bars represent mean and standard deviation. ^*^p < 0.05, ^**^p < 0.01, ^***^p < 0.0001.

In our cohort, a significant association was observed between the five prognostic factors used to define clinical profiles and the ROR score (with the exception of tumor size) or the genomic risk of recurrence (Figure 2). No correlation was observed between the ROR score or the genomic risk and the expression of estrogen or progesterone receptors (Supplementary Figure 1).

### Distribution of Intrinsic Breast Cancer Subtypes and Genomic Risk Groups Among the Clinical Profiles

**Figure 3:**
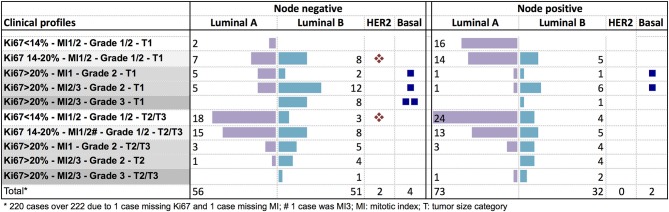
Distribution of PAM50 breast cancer subtypes according to clinical profiles in node negative or node positive (1–3 nodes) patients.

The luminal A subtype was observed across all the clinical risk groups in node negative patients. In node positive tumors, it was more frequent in tumors with Ki67 ≤ 20% (Figure 3). The luminal B subtype was less frequent in small low proliferative tumors (Ki67 < 14%).

**Figure 4:**
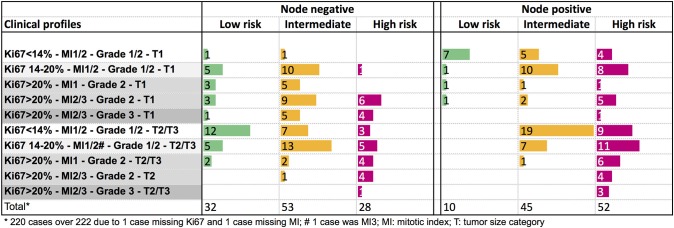
Distribution of the PAM50 risk categories according to clinical profiles in node negative or node positive (1–3 nodes) patients.

The genomic risk group of each clinical profile was described in Figure 4. In node negative tumors, no specific clinical pattern was observed for the genomic low or intermediate risk groups. The genomic high-risk group was more frequent in T2/T3 tumors, or in small tumors with Ki67 > 20%. Only 9% of node positive T1 tumors were classified as low genomic risk. The high genomic risk group was observed across all the clinical node positive profiles.

### Concordance Between Clinical Risk, Risk of Recurrence and Intrinsic Subtypes

**Figure 5:**
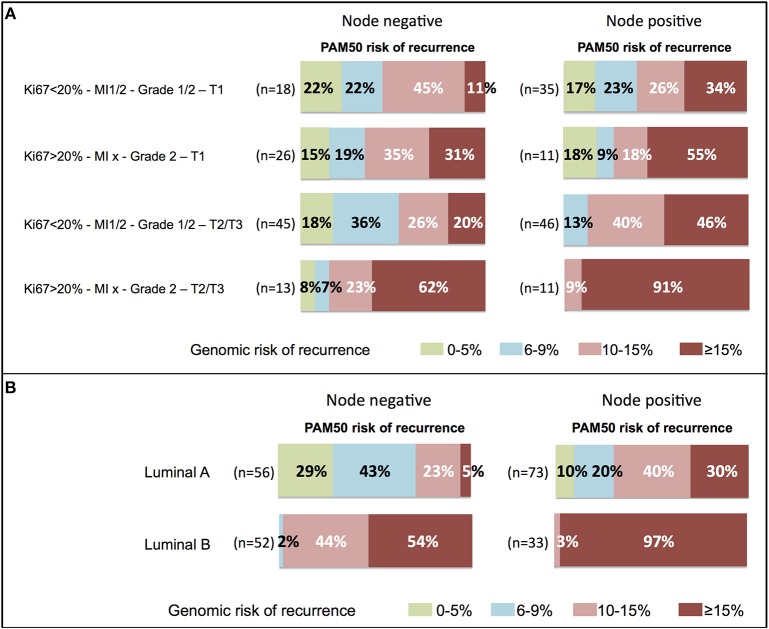
Distribution of the 10-year risk of distant recurrence estimated by PAM50 according to **(A)** clinical profiles or **(B)** luminal A or B subtypes, in node negative or node positive (1–3 nodes) patients. The range of risk was color-coded and the proportion of patients (%) indicated within the corresponding area. MI, mitotic index; T, tumor size.

In clinical high-risk patients, the genomic risk was concordant in node positive patients (1 to 3 nodes, Ki67 > 20%, grade 2, T2/T3). However, among node negative patients with these tumor characteristics, 15% had a risk of recurrence lower than 10% (Figure 5A).

Significant discordance with the genomic risk was observed among clinical low risk patients or with tumors classified as luminal A (Figure 5). 56% of the patients with node negative clinical low risk tumors (Ki67 ≤ 20%, MI 1/2, grade 1/2, T1) had a genomic risk of recurrence of 10% or more. Among patients with node positive clinical low risk tumors, 76% had a luminal A intrinsic subtype, but 60% had a genomic risk of recurrence of 10% or more (Figure 5A).

The relationship between the luminal A intrinsic subtype and the genomic risk of recurrence was analyzed in Figure 5B. Among node negative luminal A tumors, 28% had a 10% or more risk of recurrence. This proportion increased to 70% among node positive luminal A tumors.

### Impact of Clinical and Genomic Risks on Adjuvant Chemotherapy Decision

The decision whether to propose adjuvant chemotherapy or not was analyzed according to clinical profiles, genomic risk and intrinsic breast cancer subtype in node negative and node positive patients (Table 2).

**Table 2:** Decision regarding adjuvant chemotherapy according to clinical profiles, genomic risk, and intrinsic breast cancer subtype in node negative and node positive patients.

	**Luminal A**						**Luminal B**					
**Node negative (*n* = 67)**	**<10%**		**10–14%**		**>14%**		**<10%**		**10–14%**		**>14%**	
	**no CT**	**CT**	**no CT**	**CT**	**no CT**	**CT**	**no CT**	**CT**	**no CT**	**CT**	**no CT**	**CT**
Ki67 < 14%-MI1/2-G1/2-T1	1											
Ki67 14-20%-MI1/2-G1/2-T1	4		1				1		2			
Ki67>20%-MI1-G2-T1	1		1							1		
Ki67>20%-MI2/3-G2-T1	1					1			1	1		3
Ki67>20%-MI2/3-G3-T1									1	3		4
Ki67 < 14%-MI1/2-G1/2-T2/3	14		1						1			1
Ki67 14-20%-MI1/2-G1/2-T2/3	5		4	1		1				1	1^*^	2
Ki67>20%-MI1-G2-T2/3	1									1		3
Ki67>20%-MI2/3-G2-T2					1^*^							1
Ki67>20%-MI2/3-G3-T2/3												1
Total	27	0	7	1	1	2	1	0	5	7	1	15
	**Luminal A**						**Luminal B**					
**1–3 positive nodes (*****n*** **=** **84)**	**<10%**		**10–14%**		**>14%**						**>14%**	
	**no CT**	**CT**	**no CT**	**CT**	**no CT**	**CT**					**no CT**	**CT**
Ki67 < 14%-MI1/2-G1/2-T1	6		1			3						
Ki67 14–20%-MI1/2-G1/2-T1	3		6	1	1^*^	1						4
Ki67>20%-MI1-G2-T1												1
Ki67>20%-MI2/3-G2-T1	1											3
Ki67>20%-MI2/3-G3-T1												1
Ki67 < 14%-MI1/2-G1/2-T2/3	5		12	1		3						3
Ki67 14–20%-MI1/2-G1/2-T2/3			4		1#	4					1#	4
Ki67>20%-MI1-G2-T2/3			1			2					1^*^	3
Ki67>20%-MI2/3-G2-T2												4
Ki67>20%-MI2/3-G3-T2/3						1					1#	1
Total	15	0	24	2	2	14					3	24

Total of 151/159 cases in cohort 1 (Prosigna used prospectively): 8 patients excluded because classified as basal or HER2 positive by Prosigna. No chemotherapy for the following reasons: ^#^comorbidities or

* *patient refusal. CT, adjuvant chemotherapy*.

A genomic risk of recurrence < 10% was a main determinant to withhold chemotherapy, whatever the clinical profile. On the other hand, a genomic risk >14% triggered the chemotherapy decision, including in clinical low risk tumors. If the genomic risk was between 10 and 14%, the luminal A subtype was a strong factor to withhold chemotherapy. In luminal B tumors with a genomic risk between 10 and 14%, the decision to propose chemotherapy varied among oncologists.

## Discussion

A clinical assessment of the risk of recurrence and the decision with respect to the use of a multigene signature are subjective and highly variable among oncologists. Our study is the first to analyze how a multigene signature is used in a real life cohort, and its concordance with clinical risk estimates. In this cohort, the genomic signature was used mainly in clinical intermediate risk tumors. But the test was also performed in clinical high or low risk patients in case of discordant clinical and/or proliferative factors. It provided additional information about the molecular subtype or confirmed the poor prognosis in debilitated patients. Another reason for using the assay in these cases was the growing pressure from patients to have access to a multigene signature with the hope to better assess their risk of recurrence and therapeutic options.

Our study could not identify specific clinical patterns associated with genomic risk groups. However, in clinical high-risk patients, the genomic risk estimate was mostly concordant, especially in node positive patients, and did not add useful information. On the contrary, the discordance with the genomic risk was surprisingly high in node negative or node positive clinical low risk tumors (Ki67 ≤ 20%, MI 1/2, grade 1/2, T1). A significant proportion of clinical low risk patients had a ≥10% genomic risk of recurrence. These results were troublesome, particularly in node negative patients because the signature should have been omitted in these patients based on the excellent prognosis reported in the TAILORx and MINDACT trials (17, 18). In the TAILORx trial, node negative patients selected with the 21-gene signature (11–25 RS score) were mostly clinical low risk. Their 9-year distant risk of relapse was 5% and no additional benefit of chemotherapy was observed in patients over 50 years (17). The TAILORx study excluded genomic high-risk patients. In the MINDACT trial, 592 node negative patients had a low clinical risk/high genomic risk as assessed by Adjuvant Online and the 70-gene signature, respectively. In the patient population, 65% of the patients had a T1 tumor, 70% were grade 2 and 16% were grade 1; Ki67 and mitotic index were not displayed. Their distant metastasis free survival at 5 years was 4–6% (18). This prognosis and the lack of numerical difference in outcomes between the groups treated with or without chemotherapy suggest that a multigene assay in this population could be safely omitted (18, 19).

Nevertheless, in an era of personalized medicine where patients are asking for individualized care, how confidently can we extrapolate the 5-year results from MINDACT to node negative clinical low but genomic high risk patients tested with another signature? How should a clinical low risk patient with a genomic risk of 10% or more at 10 years be managed? In fact, the multigene signatures use different technical approaches and measure different genes. The PAM50-based assay used in the present study includes a higher proportion of genes involved in proliferation compared to other signatures (20), and classifies more patients in the high-risk group than the 21-gene signature (21). The comparison of several multigene signatures showed significant discrepancies both in disease subtyping and risk prediction (21, 22), highlighting their low level of concordance, which is quite problematic at an individual patient level. For this reason and until a long term follow up of MINDACT is available, extrapolations between genomic signatures should be cautious.

How confident can we be about the lack of benefit of chemotherapy in clinical low risk/ genomic high-risk node negative patients? Recently, a PAM50-based chemo-endocrine score was developed and validated to predict tumor sensitivity to treatment in the neoadjuvant setting (23). Tumors classified high-risk by the PAM50-based score or tumors with a luminal B intrinsic subtype were associated with sensitivity to chemotherapy. Based on these data, some benefit from chemotherapy cannot be excluded in clinical low risk but PAM50 high-risk patients. This patient subgroup represented almost 10% of our node negative population. This proportion may have been underestimated given that the use of the genomic signature was not systematic in clinical low risk node negative patients.

In node positive, the RxPONDER trial is ongoing (NCT01272037) to explore the benefit of adjuvant chemotherapy in 1 to 3 node positive patients selected according to their genomic risk of relapse with the 21-gene signature. Until more data is available, clinical low-risk patients may benefit from a multigene assay to adequately discuss their risk and therapeutic options.

Contrary to the study by Wallden et al. (8), our real life cohort showed that a significant proportion of tumors with an intrinsic luminal A subtype had a risk of recurrence of 10% or more. Luminal A tumors are considered to have a favorable prognosis as suggested by retrospective analyses of adjuvant trials. These studies also suggested a lack of additional benefit of chemotherapy compared to endocrine therapy (24–29). In these reports, luminal A tumors were identified based on criterion contributing to the surrogate luminal A definition (30). The ESMO 2015 guidelines recommended endocrine therapy alone for luminal A tumors, except in cases of high tumor burden (16). The St. Gallen guidelines recommended omission of adjuvant chemotherapy in stage 1 or 2 luminal A-like cancers (31). However, molecular studies characterizing the genomic and transcriptomic landscape of luminal A tumors (defined using PAM50 or genomic databases) revealed a molecular heterogeneity within this subtype, which also translated to variability in survival (32, 33). A significantly worse outcome was observed in luminal A tumors with specific molecular characteristics such as increased genomic instability, p53 alteration, amplification or overexpression of genes involved in mitosis regulation (32, 33). Whether chemotherapy could be beneficial in these high-risk luminal A tumors has not been prospectively analyzed and warrants further exploration.

Beside patients' pressure, handling of multigene signatures has proven difficult for several reasons. There is no specific recommendation to guide the decision whether these costly assays should be used. Our results showed that clinical profiles used in routine care could not reliably provide guidance. These signatures are prognostic but not predictive tools. Finally, as observed in our study, genomic risk assessment may overshadow clinical factors and become the strongest determinant for treatment decision while no randomized trial has demonstrated the superiority of a genomic driven strategy over clinical factors on patients' outcome.

Our study has some limitations. Assessment of clinical risk relied partly on proliferative markers, which have a poor reproducibility (13, 34–37). However, routine scoring of Ki67, tumor grade and mitotic index followed validated guidelines and reflected real life conditions. Moreover, these proliferative markers were highly correlated to the ROR score and the PAM50 risk of recurrence in our cohort.

In conclusion, our results showed that clinical profiles could not reliably identify genomic risk groups. The underuse of a multigene signature in clinical low risk groups may lead to inadequate risk assessment and patients' under treatment. Our study supports the use of a multigene assay in node negative or positive T1 tumors. It also highlighted a subgroup of luminal A tumors at high risk of relapse, in both node negative and node positive patients. Further work is needed to define the optimal treatment strategy in these subgroups.

## Ethics Statement

This retrospective study was carried out in accordance with the recommendations of Center Oscar Lambret Clinical Research Committee with written informed consent from all subjects. All subjects gave written informed consent in accordance with the Declaration of Helsinki. This retrospective study was approved by Center Oscar Lambret Clinical Research Committee.

## Author Contributions

NH generated the idea and study design. NH and YR performed data collection. Data analysis and writing of the manuscript was performed by NH and JB. All authors approved the final version of the manuscript.

### Conflict of Interest Statement

NH has received a speaker honorarium from NanoString Technologies and has worked as a consultant to Pfizer, Roche and Novartis. YR has received a speaker honorarium from NanoString Technologies. JB has received a speaker honorarium from NanoString Technologies and has worked as a consultant to Pfizer, Lilly, and Novartis.

## References

[1] Prat A Ellis MJ Perou CM. Practical implications of gene-expression-based assays for breast oncologists. Nat Rev Clin Oncol. (2011) 9:48–57. 10.1038/nrclinonc.2011.178 22143140 PMC3703639

[2] Harris LN Ismaila N McShane LM Andre F Collyar DE Gonzalez-Angulo AM. Use of biomarkers to guide decisions on adjuvant systemic therapy for women with early-stage invasive Breast Cancer. Am Soc Clin Oncol Clin Prac Guideline. (2016) 34:1134–50. 10.1200/JCO.2015.65.2289 26858339 PMC4933134

[3] Giuliano AE Connolly JL Edge SB Mittendorf EA Rugo HS Solin LJ. Breast Cancer-Major changes in the American Joint Committee on Cancer eighth edition cancer staging manual. CA Cancer J Clin. (2017) 67:290–303. 10.3322/caac.21393 28294295

[4] Isaacs C Stearns V Hayes DF. New prognostic factors for breast cancer recurrence. Semin Oncol. (2001) 28:53–67. 10.1053/sonc.2000.20742 11254867

[5] Gnant M Sestak I Filipits M Dowsett M Balic M Lopez-Knowles E. Identifying clinically relevant prognostic subgroups of postmenopausal women with node-positive hormone receptor-positive early-stage breast cancer treated with endocrine therapy: a combined analysis of ABCSG-8 and ATAC using the PAM50 risk of recurrence score and intrinsic subtype. Ann Oncol. (2015) 26:1685–91. 10.1093/annonc/mdv215 25935792

[6] Parker JS Mullins M Cheang MC Leung S Voduc D Vickery T. Supervised risk predictor of breast cancer based on intrinsic subtypes. J Clin Oncol. (2009) 27:1160–7. 10.1200/JCO.2008.18.1370 19204204 PMC2667820

[7] Filipits M Nielsen TO Rudas M Greil R Stöger H Jakesz R. The PAM50 risk-of-recurrence score predicts risk for late distant recurrence after endocrine therapy in postmenopausal women with endocrine-responsive early breast cancer. Clin Cancer Res. (2014) 20:1298–305. 10.1158/1078-0432.CCR-13-1845 24520097

[8] Wallden B Storhoff J Nielsen T Dowidar N Schaper C Ferree S. Development and verification of the PAM50–based Prosigna breast cancer gene signature assay. BMC Med Genomics. (2015) 8:54. 10.1186/s12920-015-0129-6 26297356 PMC4546262

[9] Bonneterre J Prat A Galván P Morel P Giard S. Value of a gene signature assay in patients with early breast cancer and intermediate risk: a single institution retrospective study. Curr Med Res Opin. (2016) 32:835–839. 10.1185/03007995.2016.1146664 26809116

[10] Allred DC Harvey JM Berardo M Clark GM. Prognostic and predictive factors in breast cancer by immunohistochemical analysis. Mod Pathol. (1998) 11:155–68. 9504686

[11] Wolff AC Hammond MEH Allison KH Harvey BE Mangu PB Bartlett JMS. Human epidermal growth factor receptor 2 testing in breast cancer: American society of clinical oncology/college of American pathologists clinical practice guideline focused update. J Clin Oncol. (2018) 36:2105–22. 10.1200/JCO.2018.77.8738 29846122

[12] Elston CW Ellis IO. Pathological prognostic factors in breast cancer. I. The value of histological grade in breast cancer: experience from a large study with long-term follow-up. Histopathology. (1991) 19:403–10 10.1111/j.1365-2559.1991.tb00229.x 1757079

[13] van Diest PJ Baak JP Matze-Cok P Wisse-Brekelmans EC van Galen CM Kurver PH. Reproducibility of mitosis counting in 2,469 breast cancer specimens: results from the Multicenter Morphometric. Mammary Carcinoma Project. Hum Pathol. (1992) 23:603–7. 10.1016/0046-8177(92)90313-R 1592381

[14] Dowsett M Nielsen TO A'Hern R Bartlett J Coombes RC Cuzick J. Assessment of Ki67 in breast cancer: recommendations from the International Ki67 in Breast Cancer working group. J Natl Cancer Inst. (2011) 103:1656–64. 10.1093/jnci/djr393 21960707 PMC3216967

[15] Goldhirsch A Winer EP Coates AS Gelber RD Piccart-Gebhart M Thürlimann B. Personalizing the treatment of women with early breast cancer: highlights of the St. Gallen International Expert Consensus on the Primary Therapy of Early Breast Cancer 2013. Ann Oncol. (2013) 24:2206–23. 10.1093/annonc/mdt303 23917950 PMC3755334

[16] Senkus E Kyriakides S Ohno S Penault-Llorca F Poortmans P Rutgers E. Primary breast cancer: ESMO clinical practice guidelines for diagnosis, treatment and follow-up. Ann Oncol. (2015) 26(Suppl. 5):v8–30. 10.1093/annonc/mdv298 26314782

[17] Sparano JA Gray RJ Makower DF Pritchard KI Albain KS Hayes DF. Adjuvant chemotherapy guided by a 21–gene expression assay in breast cancer. N Engl J Med. (2018) 379:111–121. 10.1056/NEJMoa1804710 29860917 PMC6172658

[18] Cardoso F van't Veer LJ Bogaerts J Slaets L Viale G Delaloge S. 70–Gene Signature as an Aid to treatment decisions in early-stage breast cancer. N Engl J Med. (2016) 375:717–29. 10.1056/NEJMoa1602253 27557300

[19] Krop I Ismaila N Andre F Bast RC Barlow W Collyar DE. Use of biomarkers to guide decisions on adjuvant systemic therapy for women with early-stage invasive breast cancer: American society of clinical oncology clinical practice guideline focused update. J Clin Oncol. (2017) 35:2838–47. 10.1200/JCO.2017.74.0472 28692382 PMC5846188

[20] Kwa M Makris A Esteva FJ. Clinical utility of gene-expression signatures in early stage breast cancer. Nat Rev Clin Oncol. (2017) 14:595–610. 10.1038/nrclinonc.2017.74 28561071

[21] Dowsett M Sestak I Lopez-Knowles E Sidhu K Dunbier AK Cowens JW. Comparison of PAM50 risk of recurrence score with oncotype DX and IHC4 for predicting risk of distant recurrence after endocrine therapy. J Clin Oncol. (2013) 31:2783–90. 10.1200/JCO.2012.46.1558 23816962

[22] Bartlett JM Bayani J Marshall A Dunn JA Campbell A Cunningham C. Comparing breast cancer multiparameter tests in the OPTIMA prelim trial: no test is more equal than the others. J Natl Cancer Inst. (2016) 108:djw050. 10.1093/jnci/djw050 27130929 PMC5939629

[23] Prat A Lluch A Turnbull AK Dunbier AK Calvo L Albanell J. A PAM50–Based Chemoendocrine Score for Hormone Receptor-Positive Breast Cancer with an Intermediate Risk of Relapse. Clin Cancer Res. (2017) 23:3035–44. 10.1158/1078-0432.CCR-16-2092 27903675 PMC5449267

[24] Gao JJ Swain SM. Luminal a breast cancer and molecular assays: a review. Oncologist. (2018) 23:556–65. 10.1634/theoncologist.2017-0535 29472313 PMC5947456

[25] Cheang MC Chia SK Voduc D Gao D Leung S Snider J. Ki67 index, HER2 status, and prognosis of patients with luminal B breast cancer. J Natl Cancer Inst. (2009) 101:736–50. 10.1093/jnci/djp082 19436038 PMC2684553

[26] Conforti R Boulet T Tomasic G Taranchon E Arriagada R Spielmann M. Breast cancer molecular subclassification and estrogen receptor expression to predict efficacy of adjuvant anthracyclines-based chemotherapy: a biomarker study from two randomized trials. Ann Oncol. (2007) 18:1477–1483. 10.1093/annonc/mdm209 17515403

[27] Hart CD Sanna G Siclari O Biganzoli L Di Leo A. Defining optimal duration and predicting benefit from chemotherapy in patients with luminal-like subtypes. Breast. (2015) 24:S136–42. 10.1016/j.breast.2015.07.033 26320761

[28] Coates AS Colleoni M Goldhirsch A. Is adjuvant chemotherapy useful for women with luminal a breast cancer? J Clin Oncol. (2012) 30:1260–3. 10.1200/JCO.2011.37.7879 22355052

[29] Coates AS Winer EP Goldhirsch A Gelber RD Gnant M Piccart-Gebhart M.. Tailoring therapies – improving the management of early breast cancer: St. Gallen International Expert Consensus on the Primary Therapy of Early Breast Cancer. Ann Oncol. (2015) 26:1533–46. 10.1093/annonc/mdv221 25939896 PMC4511219

[30] Goldhirsch A Wood WC Coates AS Gelber RD Thürlimann B Senn HJ. Strategies for subtypes–dealing with the diversity of breast cancer: highlights of the St. Gallen International Expert Consensus on the Primary Therapy of Early Breast Cancer 2011. Ann Oncol. (2011) 22:1736–47. 10.1093/annonc/mdr304 21709140 PMC3144634

[31] Curigliano G Burstein HJ Winner EP Gnant M Dubsky P Loibl S. De-escalating and escalating treatments for early-stage breast cancer: the St. Gallen International Expert Consensus Conference on the Primary Therapy of Early Breast Cancer 2017. Ann Oncol. (2017) 28:1700–12. 10.1093/annonc/mdx308 28838210 PMC6246241

[32] Ciriello G Sinha R Hoadley KA Jacobsen AS Reva B Perou CM. The molecular diversity of Luminal A breast tumors. Breast Cancer Res Treat. (2013) 141:409–20. 10.1007/s10549-013-2699-3 24096568 PMC3824397

[33] Pérez-Peña J Alcaraz-Sanabria A Nieto-Jiménez C Páez R Corrales-Sánchez V Serrano-Oviedo L. Mitotic read-out genes confer poor outcome in luminal A breast cancer tumors. Oncotarget. (2017) 8:21733–40. 10.18632/oncotarget.15562 28423514 PMC5400619

[34] Laenkholm AV Grabau D Møller Talman ML Balslev E Bak Jylling AM Tabor TP. An inter-observer Ki67 reproducibility study applying two different assessment methods: on behalf of the Danish Scientific Committee of Pathology, Danish breast cancer cooperative group (DBCG). Acta Oncol. (2018) 57:83–9. 10.1080/0284186X.2017.1404127 29202622

[35] Penault-Llorca F Radosevic-Robin N. Ki67 assessment in breast cancer: an update. Pathology. (2017) 49:166–71. 10.1016/j.pathol.2016.11.006 28065411

[36] Rimm DL Leung SCY McShane LM Bai Y Bane AL Bartlett JMS. An international multicenter study to evaluate reproducibility of automated scoring for assessment of Ki67 in breast cancer. Mod Pathol. (2018) 32:59–69. 10.1038/s41379-018-0109-4 30143750

[37] Skaland I van Diest PJ Janssen EA Gudlaugsson E Baak JP. Prognostic differences of World Health Organization-assessed mitotic activity index and mitotic impression by quick scanning in invasive ductal breast cancer patients younger than 55 years. Hum Pathol. (2008) 39:584–90. 10.1016/j.humpath.2007.08.016 18291440

