# Supplementary material for: Should a Multigene Signature be Used in all Luminal Early Breast Cancers
Source: Front Oncol. 2019 Jun 4;9:454. doi: 10.3389/fonc.2019.00454 (PMC6558017; doi:10.3389/fonc.2019.00454)
Supplement: Supplementary file 1 [file Data_Sheet_1.docx]

Supplementary Material

# Supplementary Figures and Tables

## Supplementary Table 1

Patients and tumor characteristics of the two-pooled cohorts

|  |  |  |  | Prospective Cohort (n=159) | |  | Retrospective  Cohort (n=63) | | | |  | |  |
| --- | --- | --- | --- | --- | --- | --- | --- | --- | --- | --- | --- | --- | --- |
|  |  |  |  | n | % |  | n | % | | |  | | p |
| Age (median, range) |  |  |  | 55 | (29-81) | | 61 | (50-77) | | |  | | 0.0002 |
| Body Mass Index | |  |  | 25.56 (17.40 – 54.43) | | | 26.56 (16.96-43.42) | | | | | | 0.71 |
| Tumor size (mm, median, range) | |  |  | 22 | (3-100) | | 20 | (2-55) | | |  | | 0.02 |
| T1 |  |  |  | 66 | 41 |  | 36 | 57 | |  | |  | |
| T2 |  |  |  | 76 | 48 |  | 26 | 41 | |  | |  | |
| T3 |  |  |  | 17 | 11 |  | 1 | 2 | |  | |  | |
| Lumpectomy |  |  |  | 89 | 56 |  | 47 | 75 | |  | | 0.01 | |
| Mastectomy |  |  |  | 70 | 44 |  | 16 | 25 | |  | |  | |
| Axillary exploration | | |  |  |  |  |  |  | |  | | 0.51 | |
| Sentinel lymph node |  |  |  | 96 | 60 |  | 37 | 59 | |  | |  | |
| Axillary dissection |  |  |  | 60 | 38 |  | 26 | 41 | |  | |  | |
| None |  |  |  | 3 | 2 |  | _ | _ | |  | |  | |
| Histology |  |  |  |  |  |  |  |  | |  | | 0.11 | |
| Ductal |  |  |  | 104 | 65 |  | 51 | 81 | |  | |  | |
| Lobular |  |  |  | 36 | 23 |  | 7 | 11 | |  | |  | |
| Other |  |  |  | 19 | 12 |  | 5 | 8 |  | | | |  |
| Grade |  |  |  |  |  |  |  |  |  | | | | 0.04 |
| 1 |  |  |  | 14 | 9 |  | 6 | 10 |  | | | |  |
| 2 |  |  |  | 130 | 82 |  | 57 | 90 |  | | | |  |
| 3 |  |  |  | 15 | 9 |  | _ | _ |  | | | |  |
| Ki67 |  |  |  |  |  |  |  |  |  | | | | 0.56 |
| <14% |  |  |  | 52 | 33 |  | 16 | 25 |  | | | |  |
| 14-20% |  |  |  | 55 | 35 |  | 22 | 35 |  | | | |  |
| >20% |  |  |  | 52 | 33 |  | 24 | 38 |  | | | |  |
| n |  |  |  | _ | _ |  | 1 | 2 |  | | | |  |
| Mitotic index |  |  |  |  |  |  |  |  |  | | | | 0.0001 |
| 1 |  |  |  | 109 | 69 |  | 27 | 43 |  | | | |  |
| 2 |  |  |  | 40 | 25 |  | 34 | 54 |  | | | |  |
| 3 |  |  |  | 10 | 6 |  | 1 | 2 |  | | | |  |
| n |  |  |  | _ | _ |  | 1 | 2 |  | | | |  |
| Estrogen receptor positive | |  |  | 153 | 96 |  | 63 | 100 |  | | | | 0.80 |
| Progesterone receptor positive | | |  | 132 | 83 |  | 52 | 83 |  | | | | 0.83 |
| HER2 status negative |  |  |  | 159 | 100 |  | 63 | 100 |  | | | | ns |
| Nodal involvement |  |  |  |  |  |  |  |  |  | | | | 0.16 |
| 0 |  |  |  | 72 | 45 |  | 41 | 65 |  | | | |  |
| 1 |  |  |  | 49 | 31 |  | 14 | 22 |  | | | |  |
| 2 |  |  |  | 29 | 18 |  | 7 | 11 |  | | | |  |
| 3 |  |  |  | 9 | 6 |  | 1 | 2 |  | | | |  |
|  |  |  |  |  |  |  |  |  |  | | | |  |

## Supplementary Figure 1

**Supplementary Figure 1.** Correlation between the ROR score or the PAM50 risk of recurrence and progesterone receptor (A, C), or estrogen receptor (B, D) expression. Results were not significant by Spearman test
